# A novel lytic phage exhibiting a remarkable in vivo therapeutic potential and higher antibiofilm activity against *Pseudomonas aeruginosa*

**DOI:** 10.1007/s10096-023-04649-y

**Published:** 2023-08-23

**Authors:** Aliaa Abdelghafar, Amira El-Ganiny, Ghada Shaker, Momen Askoura

**Affiliations:** Department of Microbiology and Immunology, Faculty of Pharmacy, Zagazig University, Zagazig, 44519 Egypt

**Keywords:** *Pseudomonas aeruginosa*, Phage therapy, *Myoviridae*, Sequencing

## Abstract

**Background:**

*Pseudomonas aeruginosa* is a nosocomial bacterium responsible for variety of infections. Inappropriate use of antibiotics could lead to emergence of multidrug-resistant (MDR) *P. aeruginosa* strains. Herein, a virulent phage; vB_PaeM_PS3 was isolated and tested for its application as alternative to antibiotics for controlling *P. aeruginosa* infections.

**Methods:**

Phage morphology was observed using transmission electron microscopy (TEM). The phage host range and efficiency of plating (EOP) in addition to phage stability were analyzed. One-step growth curve was performed to detect phage growth kinetics. The impact of isolated phage on planktonic cells and biofilms was assessed. The phage genome was sequenced. Finally, the therapeutic potential of vB_PaeM_PS3 was determined in vivo.

**Results:**

Isolated phage has an icosahedral head and a contractile tail and was assigned to the family *Myoviridae*. The phage vB_PaeM_PS3 displayed a broad host range, strong bacteriolytic ability, and higher environmental stability. Isolated phage showed a short latent period and large burst size. Importantly, the phage vB_PaeM_PS3 effectively eradicated bacterial biofilms. The genome of vB_PaeM_PS3 consists of 93,922 bp of dsDNA with 49.39% G + C content. It contains 171 predicted open reading frames (ORFs) and 14 genes as tRNA. Interestingly, the phage vB_PaeM_PS3 significantly attenuated *P. aeruginosa* virulence in host where the survival of bacteria-infected mice was markedly enhanced following phage treatment. Moreover, the colonizing capability of *P. aeruginosa* was markedly impaired in phage-treated mice as compared to untreated infected mice.

**Conclusion:**

Based on these findings, isolated phage vB_PaeM_PS3 could be potentially considered for treating of *P. aeruginosa* infections.

**Supplementary Information:**

The online version contains supplementary material available at 10.1007/s10096-023-04649-y.

## Background

*Pseudomonas aeruginosa* is a widely distributed opportunistic pathogen that could survive in water, soil, animals and humans [1]. *P. aeruginosa* causes a variety of acute and chronic infections especially in immunocompromised patients [2, 3]. *P. aeruginosa* infections are very difficult to eradicate owing to higher resistance to antibiotics [4]. *P. aeruginosa* has been recently listed as a multidrug resistant (MDR) pathogen by the World Health Organization (WHO) and represents one of the biggest threats to public health [5]. This resistance could be related to bacterial inherited antibiotic resistance or to the acquisition of resistance genes through mobile genetic elements [4]. Additionally, it has been suggested that biofilm formation markedly contributes to bacterial resistance to various antibiotics, including quinolones, aminoglycosides and β-lactams leading to long term persistence [6, 7]. Biofilms confer higher antibiotic resistance than planktonic cells due to diminished antibiotic penetration through extracellular polymeric matrix in addition to the lower metabolic activity of persister cells [8, 9]. Therefore, there is a critical demand to develop new approaches to inhibit *P. aeruginosa* biofilms and keep this pathogen under control. Latterly, researchers have indicated that phage therapy could be considered as an effective approach to eradicate *P. aeruginosa* biofilms [10, 11].

Bacteriophages also known informally as phages are viruses that selectively infect and replicate within bacterial cells and considered the most prevalent entities on the earth [12]. Phage therapy refers to the use of bacteriophages in treatment of bacterial infections as potential alternatives to overcome limitations of antibiotics [13]. Phages possess remarkable advantages over antibiotics and are extremely specific to their host bacteria as well as they do not cause harmful side effects to normal microbiota [14]. In addition, phages are easily isolated and highly effective at destroying bacteria in biofilms [15, 16].

Unlike chemical antibiotics, phages have different strategies for eliminating resistant bacteria and have low environmental impact due to their natural origin [17, 18]. An important feature of phages is their specificity of action, exhibiting a narrow spectrum of activity. Furthermore, many reports have shown that phages are highly effective against various MDR pathogens in humans and animals [19, 20]. Hence, phage therapy possesses a great potential to replace antibiotic treatment in treatment of various bacterial infections [21].

While many previous studies have reported isolation of a huge number of phages infecting *P. aeruginosa,* there is still a tremendous lack of knowledge regarding either genomic characterization [22, 23], the antibiofilm activity or host safety of isolated phages targeting *P. aeruginosa* [24–28]. Hence, the present research is directed to introduce and widely characterize a virulent phage infecting *P. aeruginosa*. The phage genome, physical characteristics, environmental stability as well as antibiofilm activity will be uncovered in the present study. Finally, the capacity of isolated phage to limit *P. aeruginosa* virulence potential in host will be evaluated in vivo. The outcomes of current study are expected to be valuable for establishing effective treatment and controlling infectious diseases caused by *P. aeruginosa*.

## Material and methods

### Bacterial strains

Fifteen *P. aeruginosa* strains isolated from various clinical sources were provided by laboratories at the University Hospital, Zagazig, Egypt, with no direct involvement of patients in the study. In addition, *P. aeruginosa* reference strains, PAO1, ATCC 9027, and ATCC 27853, were involved in present study (Table 1).

**Table 1:** Host range and efficiency of plating (EOP) of phage vB_PaeM_PS3

Bacterial isolate ^a^	Infectivity ^b^	EOP ratio (Mean ± SD)	Interpretation
PS 1B	** + **	0.41 ± 0.04	Medium
PS 2B	**-**	-	-
PS 3B	** + **	1	High (host)
PS 4B	**-**	-	-
PS 5W	**-**	-	-
PS 6W	** + **	0.042 ± 0.005	Low
PS 7W	** + **	0.8 ± 0.03	High
PS 8U	**-**	-	-
PS 9U	**-**	-	-
PS 10U	** + **	0.61 ± 0.05	High
PS 11U	** + **	0.02 ± 0.003	Low
PS 12SP	**-**	-	-
PS 13SP	** + **	0.75 ± 0.07	High
PS 14SP	**-**	-	-
PS 15E	** + **	1.23 ± 0.07	High
*P. aeruginosa* PAO1	** + **	0.034 ± 0.005	Low
*P. aeruginosa* ATCC 27853	** + **	0.28 ± 0.03	Medium
*P. aeruginosa* ATCC 9027	**-**	-	-
*E. coli* ATCC 10536	**-**	-	-
*E. coli* ATCC O26	**-**	-	-
*E. coli* ATCC O78	**-**	-	-
*E. coli* ATCC 0157	**-**	-	-
*S. Typhimurium* ATCC 14028	**-**	-	-
*K. pneumoniae* ATCC 700603	**-**	-	-
*Serratia marcescens*	**-**	-	-
*S. aureus* ATCC 6538	**-**	-	-
*S. aureus* ATCC 9295	**-**	-	-

^**a**^B; burn, W; surgical wound, U; urine, SP; endotracheal aspirates

^**b**^ + : indicates presence of clear zone (lysis) and -: indicates no lysis was observed

### Bacteriophage isolation, purification, and propagation

Wastewater samples were collected from Zagazig city, Egypt, for phage isolation by enrichment procedure as described previously [29]. Briefly, wastewater samples were centrifugated (6000 × *g*, 10 min), then the supernatant was filtrated through a 0.4-μm sterilized syringe filter. *P. aeruginosa* was grown to exponential phase, infected with the obtained filtrate in double concentrated tryptone soya (TS) broth then incubated with shaking for 24 h at 37°C. Afterwards, the enriched culture was centrifuged, filtered using 0.22 µm filter, then spot assay was performed to detect presence of phages in the filtrate as described [30]. Phage purification was performed using double agar layer method as described before [31]. Phage propagation was carried out according to Gencay et al. [32] by adding 5 mL of phage SM buffer to plates with confluent lysis, left overnight at 4°C and filtered by a 0.22 filter. Finally, the phages were kept at higher titer in a refrigerator at 4°C [32].

### Transmission electron microscopy (TEM)

The morphology of phage particles was determined using transmission electron microscope (Hitachi H600A, Japan) exactly as previously described following staining with 2% phosphotungstic acid [33].

### Host range determination and efficiency of plating (EOP) analysis

Host range specificity of isolated phage was determined using standard spot assay [34]. Bacteriophage efficiency of plating was determined by double agar layer technique. Briefly, tenfold serially diluted phage (100 µL) was mixed with exponential phase bacterial culture (100 µL) in soft agar layer and poured over the surface of TS agar plates followed by incubation at 37°C. The plaque forming units (PFUs) were counted for susceptible strains. The relative EOP values were determined by calculating the ratio of phage titer for a given bacteria to phage titer of the relevant host bacteria [35].

### Phage stability to environmental conditions

Thermal stability of isolated phage was assessed by incubating phage particles at various temperatures (4–100°C) for 1 h. Similarly, phage pH stability was evaluated by incubating phage for 1 h in SM buffer adjusted at wide pH range (3–12) adjusted by 1 M HCl or 1 M NaOH. The number of surviving phages was determined by the double agar layer technique from triplicate assays [36].

### Phage growth kinetics analysis

One-step growth curve for isolated phage was carried out to detect phage burst size and latent period as described [37]. Initially, bacterial host strain was cultured to reach exponential phase [10^8^ colony forming units (CFU)/mL] and mixed with isolated phage at multiplicity of infection (MOI) of 0.1. Then, the mixture was centrifuged for 10 min at 10000 × *g*, pellets were resuspended in fresh TS broth and incubated at 37°C. Simultaneously, phage titer was determined by collecting samples of 100 µL at 5 min intervals then plated by double agar layer method.

### In vitro killing assay and Biofilm inhibition assay

In vitro bacteriolytic activity of isolated phage was determined by measuring optical density (OD_600_) as mentioned before [38]. The phage was added to exponential phase bacterial cultures at different MOIs (0.1, 1, and 10). The phage/bacteria mixture was incubated at 37°C for 24 h. Bacterial culture without phage was used as a control. The phage-induced bacterial lysis (bacterial growth inhibition) was observed by measuring change in OD_600_. The antibiofilm activity of isolated phage was determined as exactly as previously described [39]. The phage was diluted in sterile TS broth to reach different MOIs (0.1, 1 and 10), then added to wells containing preformed bacterial biofilms followed by incubation at 37°C for 24 h. Control wells received an equivalent amount of TS broth only. The biomass of formed biofilms was quantified spectrophotometrically by the crystal violet technique using a microplate reader (Biotek, USA).

### Determination of the frequency of bacteriophage insensitive mutants (BIMs)

The frequency of occurrence of bacteriophage insensitive mutants (BIMs) was determined as previously described [40]. Briefly, bacterial host culture was mixed with the phage suspension at MOI of 100 and incubated for 10 min at 37°C. Then, 100 µL of phage-bacterium mixture was serially diluted, plated on TS agar and incubated overnight at 37°C. The number of resulting colonies was counted from triplicate assays and the BIM frequency was estimated by dividing surviving bacterial colonies by initial viable counts and results were expressed as means ± standard deviation.

### Phage-antibiotic synergy assay

The synergetic effect between isolated phage and two antibiotics commonly used for *pseudomonas* infections and represent different antibiotic classes, gentamicin (aminoglycoside) and ciprofloxacin (quinolone), was determined using checkerboard microdilution assay by calculating the fractional inhibitory concentration index (FICI) [41]. Briefly, the antibiotic was two-fold serially diluted and the vB_PaeM_PS3 phage was tenfold serially diluted followed by addition of bacterial suspension (10^5^ CFU/mL) to each well of 96-well plate. The plates were incubated overnight at 37°C. The FICI was calculated using the following equation: FICI = FIC antibiotic + FIC phage; FIC antibiotic = C_antibiotic_ / MIC_antibiotic_; FIC phage = C_phage_ / MIC_phage_, where MIC_antibiotic_ and MIC_phage_ are the respective minimum inhibitory concentration (MICs) of the antibiotic and the phage alone, and C_antibiotic_ and C_phage_ are the respective concentrations of the antibiotic and phage in combination. The results were interpreted as the follow; synergy if FICI was ≤ 0.5; indifferent if 0.5 ˂ FICI ≤ 1; additive if 1 ˂ FIC ≤ 2 and antagonistic if FICI > 2.

### Bacteriophage genome sequencing and data analysis

The genome of isolated phage was extracted using QIAamp1 DNA Mini kit (QIAGEN, Germany) following the manufacturer guidelines. The Nextera XT DNA Library Preparation Kit (Illumina, USA) was used to prepare the DNA library. The phage genome was sequenced at Genomics and Epigenomics Program, Cairo, Egypt, using Illumina Miseq next-generation sequencing. The raw sequence within the phage genome was checked for quality with FastQC and reads trimming was performed using Trimmomatic v0.36 [42]. The trimmed reads were assembled de novo into a single contig using Unicycler v0.4.8 [43]. The assembly quality was checked with Quality Assessment Tool for Genome Assemblies (QUAST—v4.4) [44]. Sequence analysis and annotation of resulting open reading frames (ORFs) were predicted using Prokka v1.14 [45] and Rapid Annotation Subsystems Technology (RAST) [46]. The virus circular genome map was created using the CGView [47]. Transfer RNA was detected using the online tool tRNAscan-SE 1.21 [48]. The phylogenetic analysis of phage whole genome was performed using the MEGA X program v10.2.2. [49]. Additionally, the nucleotide sequences of both the phage major capsid protein and terminase large subunit genes were compared with their corresponding sequences of reference bacteriophages deposited in the NCBI database to determine the phylogenetic position of the recently isolated phage [50]. Comparison of phage genome with similar phages was accomplished using the EasyFig program [51]. A dot plot was constructed using the Gepard-2.1 [52]. The annotated genome sequence of isolated phage was submitted to the National Center for Biotechnology Information (NCBI) nucleotide database and deposited in the GenBank under accession number (GenBank Acc. No OQ411628).

### In vivo mice infection assay

The impact of phage on *Pseudomonas* infectivity was investigated in vivo using mice [26]. Briefly, five mice groups; each one contains 15 mice were included in the experiment. In the 1^st^ group, mice were infected intraperitoneally (IP) with *P. aeruginosa* (2.5 × 10^7^ CFU/mL), while in the 2^nd^ group, mice were infected with *P. aeruginosa* and treated IP with phage at MOI of 100 (2.5 × 10^9^ PFU/mL). The 3^rd^ group represents mice without bacterial infection but inoculated only with phage. As negative controls, mice injected with PBS only and non-injected mice were included in the experiment. Mice survival was monitored and statistically analysed using Log-rank test. Additionally, three mice from each group were subjected to bacterial load and phage titer determination. Mice spleen and liver were collected, then bacterial burden as well as phage titer were quantified and statistically analyzed with *P* value < 0.05 is considered significant.

## Results

### Isolation and characterization of phage

**Fig. 1:**
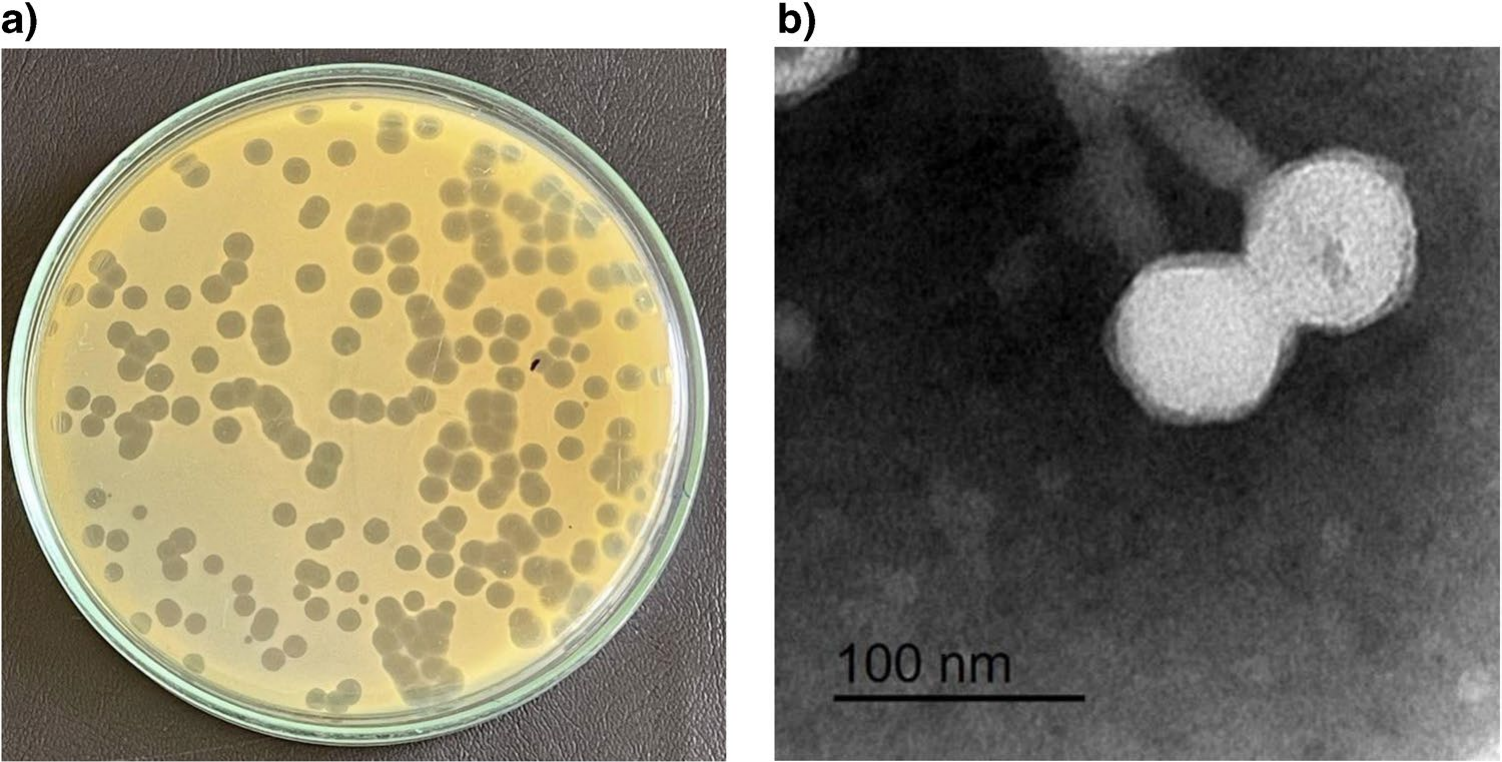
**a**) Plaque morphology of vB_PaeM_PS3 on *P. aeruginosa* lawn by double agar layer technique. **b**) Transmission electron microscopy (TEM) of negatively stained phage particles. Scale bar = 100 nm

The phage was isolated from wastewater sample using *P. aeruginosa* PS3 as a host. Isolated phage produced clear circular plaques surrounded by halos with a diameter of 4–5 mm (Fig. 1a). Phage particles morphology were observed using TEM revealing that isolated phage has an icosahedral head of about 70 nm in diameter and a contractile tail of 100 nm in length (Fig. 1b). The phage was classified according guidelines of International Committee Taxonomy of Viruses (ICTV) and found to belong to the order *Caudovirales* and the family *Myoviridae*. The phage isolated herein was named as vB_PaeM_PS3 following the phage nomenclature recommendation [53].

### Determination of host range and efficiency of plating (EOP)

The host range specificity of vB_PaeM_PS3 lysate was examined using the spot assay. Results show that isolated phage has a unique lytic profile and was able to infect 10 out of 18 of tested *P. aeruginosa* strains (55.5%) indicating a higher lytic efficiency of isolated phage (Table 1). To further assess the phage vB_PaeM_PS3 infection capability, EOP analysis was performed for each susceptible strain. vB_PaeM_PS3 phage was able to infect and form plaques (produce new progeny) on all susceptible *P. aeruginosa* strains with different infectivity patterns. Results show that only one *P. aeruginosa* isolate; PS15 exhibits efficient production higher than host strain (Table 1).

### Environmental stability and one-step growth curve

**Fig. 2:**
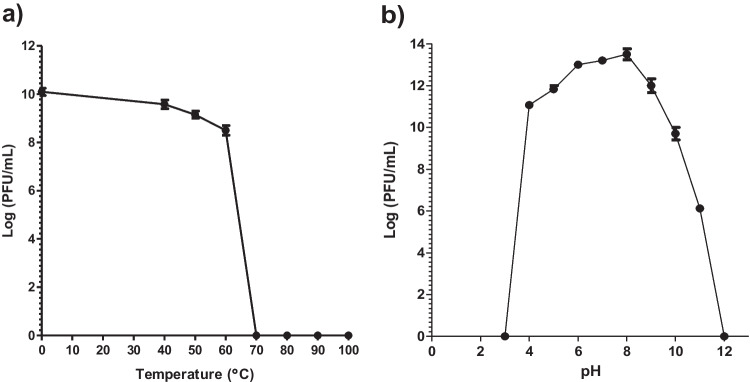
Stability analysis of vB_PaeM_PS3. Phage incubated for 1 h at different temperatures (**a**) and pH values (**b**). Plaques were counted and the results were expressed as mean ± SE for three independent experiments

**Fig. 3:**
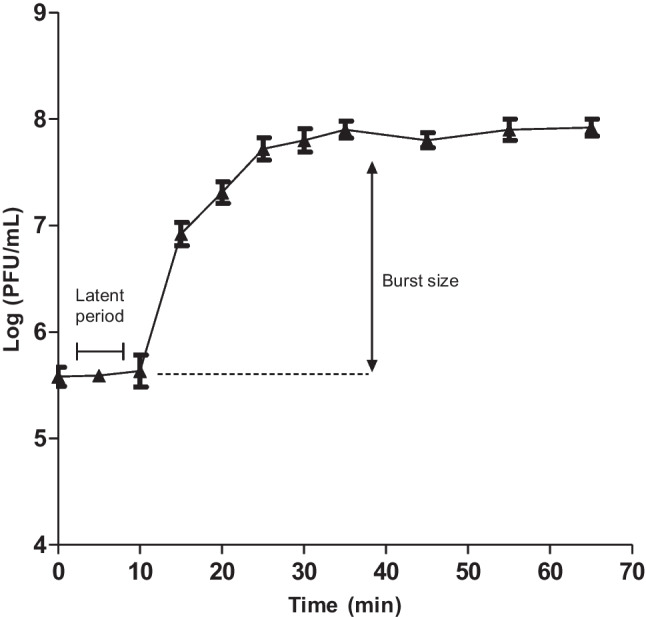
One step growth curve of vB_PaeM_PS3. Free phages were counted at 5-min interval by double layer agar technique. The experiment was repeated three times and data are expressed as mean ± SE

Sensitivity of vB_PaeM_PS3 to different environmental conditions was characterized and phage survival under various temperatures and pH values was assessed. As shown in Fig. 2a, the phage vB_PaeM_PS3 was considerably stable at temperatures range from 40 to 60°C. However, the activity of vB_PaeM_PS3 was entirely diminished when incubated at 70–100°C. Additionally, the vB_PaeM_PS3 titer remained relatively unchanged when incubated at pH values ranging from 5 to 10. However, the phage infectivity slightly decreased at pH 11 and completely lost at pH 3 & 12 as shown in Fig. 2b. The one-step growth curve was used to characterize vB_PaeM_PS3 growth characters. The phage vB_PaeM_PS3 exhibited a short latent period of 10 min with host-cell lysis releasing about of 132 new virions per infected cell (Fig. 3).

### In vitro killing and biofilm degradation assay

**Fig. 4:**
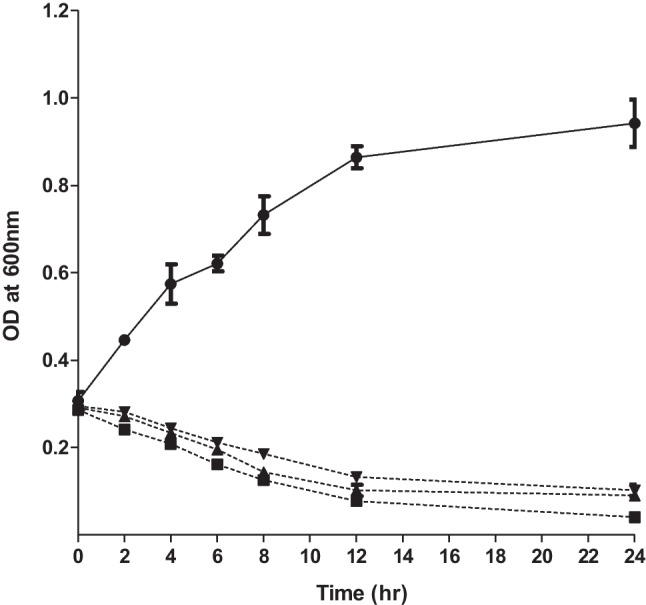
In vitro planktonic cell lysis assay of vB_PaeM_PS3 at different MOIs against host bacteria. Bacterial growth inhibition was measured spectrophotometrically (OD_600_) for bacterial cultures with and without phage over 24 h. The results were expressed as means ± SE of three independent experiments

**Fig. 5:**
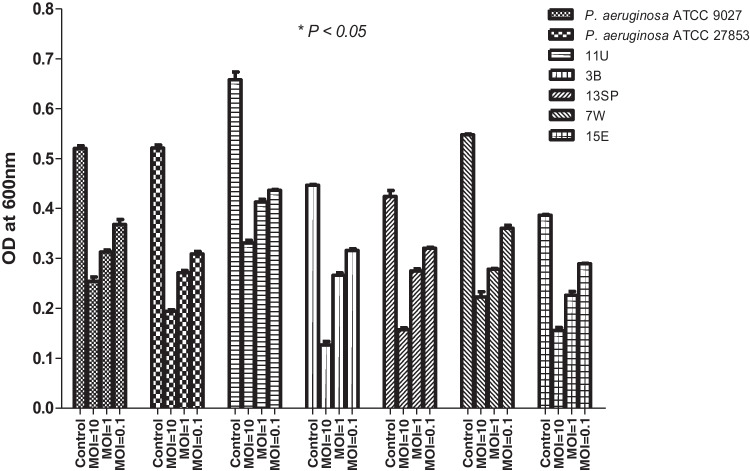
Effect of vB_PaeM_PS3 phage treatment on *P. aeruginosa* biofilms. Bacterial isolates were allowed to form biofilms for 24 h then treated with phage at various MOIs (0.1, 1 & 10) for 24 h. Biofilm biomass was evaluated by crystal violet (CV) staining. Data was expressed as means ± SE from three independent replicates with *P* < 0.05 was considered significant

The bacteriolytic activity of vB_PaeM_PS3 phage was determined in broth culture medium. *P. aeruginosa* host strain (PS3) was infected with vB_PaeM_PS3 phage at various MOIs (0.1, 1 and 10). As shown in Fig. 4, growth of vB_PaeM_PS3-infected *P. aeruginosa* significantly decreased compared to the control *P. aeruginosa* culture without phage treatment. Remarkably, the cell lysis capacity of the phage was MOI dependent and bacterial inhibition was more obvious at higher MOIs. Furthermore, the ability of vB_PaeM_PS3 to eradicate biofilms of five clinical *P. aeruginosa* isolates recovered from various sources as well as the reference strains; ATCC 27853 and 9027 was evaluated. The phage vB_PaeM_PS3 showed a remarkable biofilm degrading efficiency both at higher and lower MOIs (MOI of 10 and 0.1, respectively) (Fig. 5). In addition, there was a considerable decrease in bacterial biomass compared to the control phage-untreated bacteria.

### Frequency of BIMs

The frequency of emergence of BIMs is considered as one of the major important characteristics for phage therapy. It is worth noting that the mutation frequency parameter in the host strain was [1.8 ± 0.1] × 10^–3^. This result indicates low ability to develop phage-resistance mutants and long-term effectiveness using phage therapy confirming that vB_PaeM_PS3 phage would be a promising candidate for phage therapy in the future.

### The phage vB_PaeM_PS3 exhibits a synergistic effect on commonly used antibiotics

To establish the synergistic effect of vB_PaeM_PS3 phage in combination with conventional used antibiotics; gentamicin and ciprofloxacin, the FICIs values were determined. Interestingly, the MICs values for gentamicin and ciprofloxacin were markedly reduced when combined with vB_PaeM_PS3 phage giving FIC values of 0.16 and 0.26, respectively which were lower than 0.5 indicating a synergistic effect. MICs values of gentamicin and ciprofloxacin dropped from 32 and 16 to 2 and 4 µg / mL, respectively. These results clearly suggest that isolated phage could be used in combination with antibiotics to effectively control *P. aeruginosa* infections.

### Phage genome analysis

**Fig. 6:**
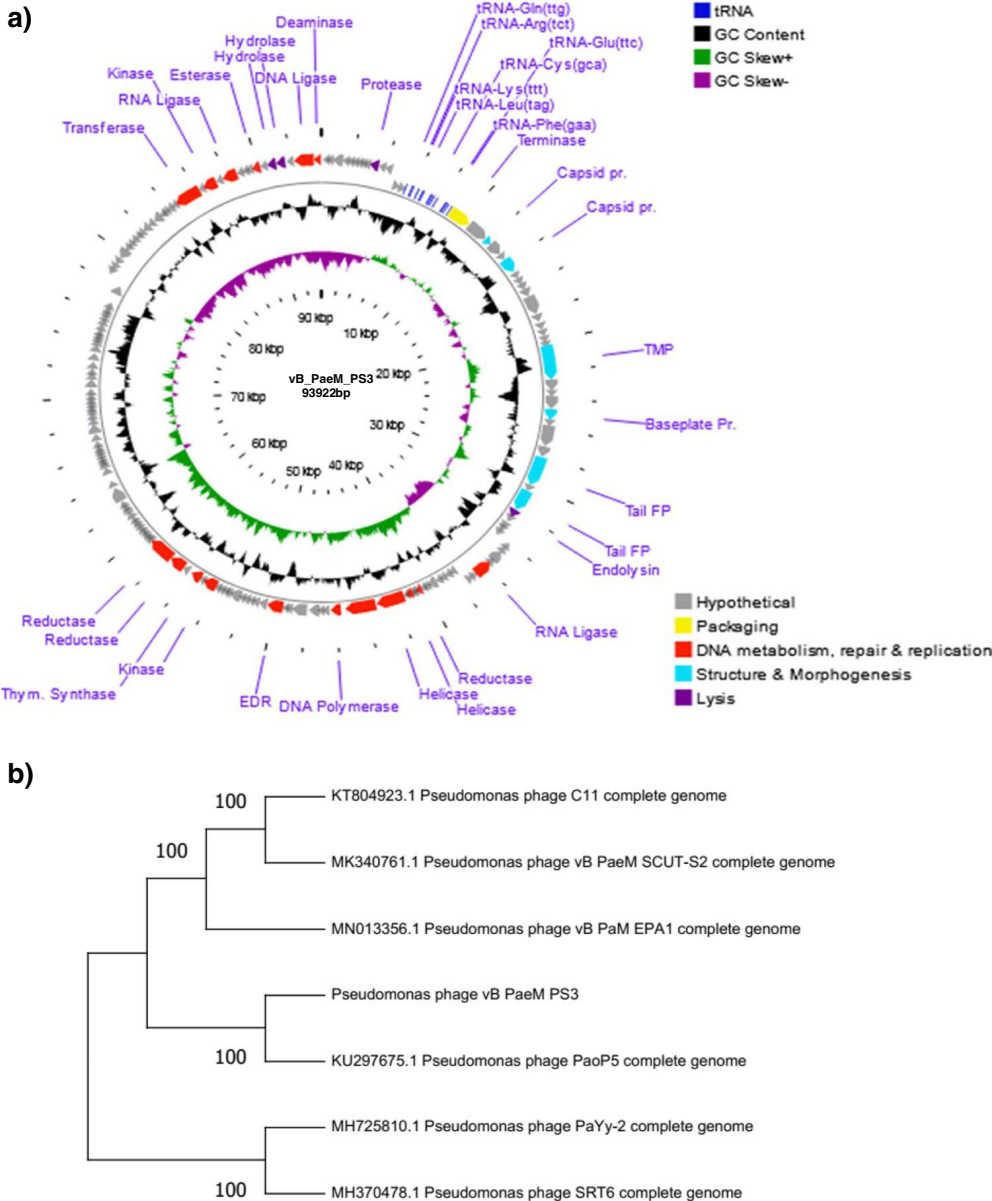

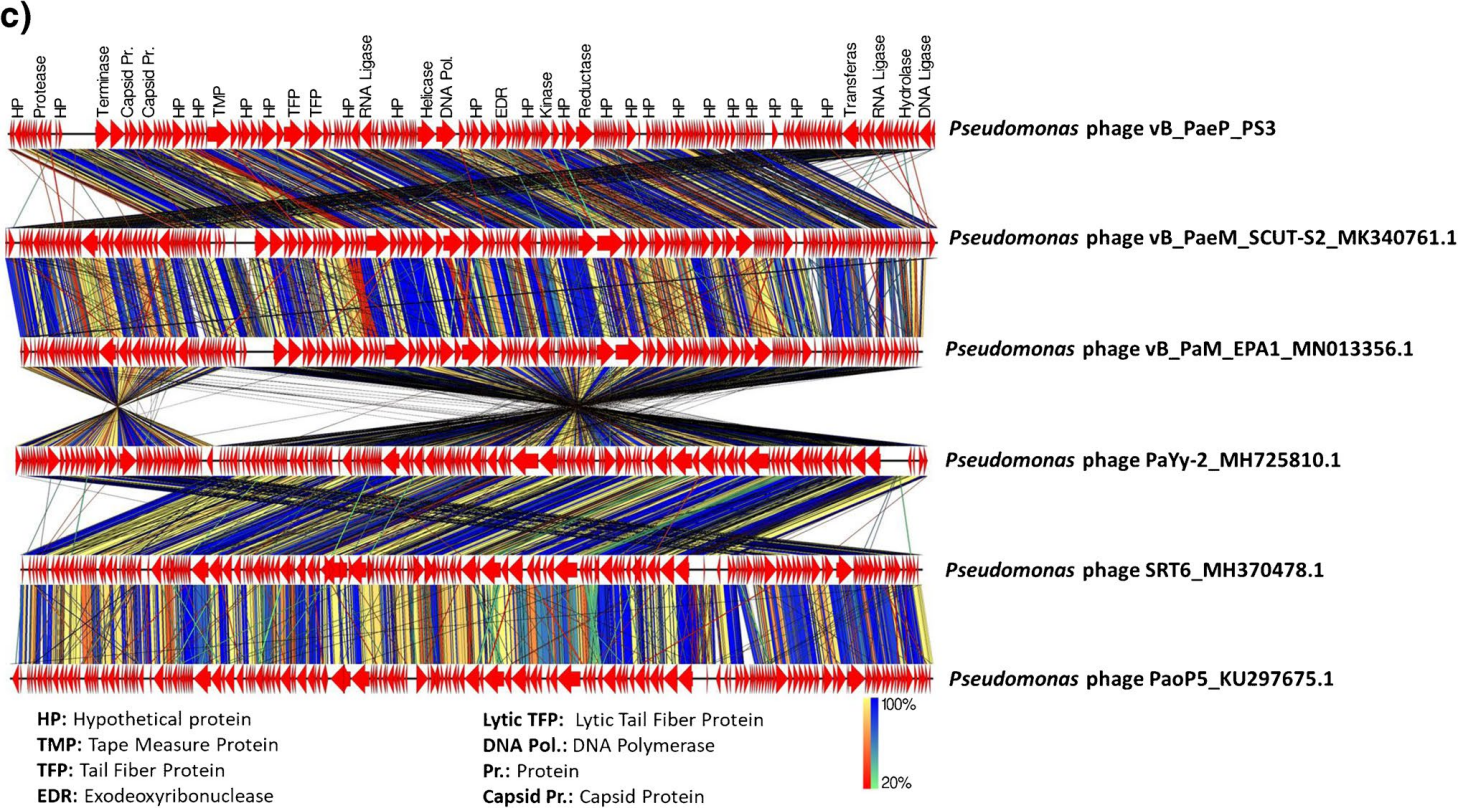
General features of vB_PaeM_PS3 genome. (**a**) Schematic genomic map of vB_PaeM_PS3 phage. The inner rings represent genome location, GC skew + (green) and GC skew (purple) and GC content (black). ORFs are represented by colored arrows. Functional ORFs were classified into four groups: yellow; DNA packaging, red; DNA metabolism, repair and replication, blue; structural proteins and purple; lysis proteins. Hypothetical proteins are indicated in grey. The figure was generated using CGView program. (**b**) Phylogenetic tree of vB_PaeM_PS3 using whole genome sequence and other closely related sequences. Phylogenetic tree was generated using MEGA-X computer program. (**c**) Comparative genomic analysis of the phage vB_PaeM_PS3 with homologous phages. Similarity level among phage sequences is represented by the colored scale bar from 20 to 100%. The coding sequences are represented by directional arrows. Predicted ORFs in vB_PaeM_PS3 genome are listed below. Genomic comparison was performed and plotted using Easyfig program

**Fig. 7:**
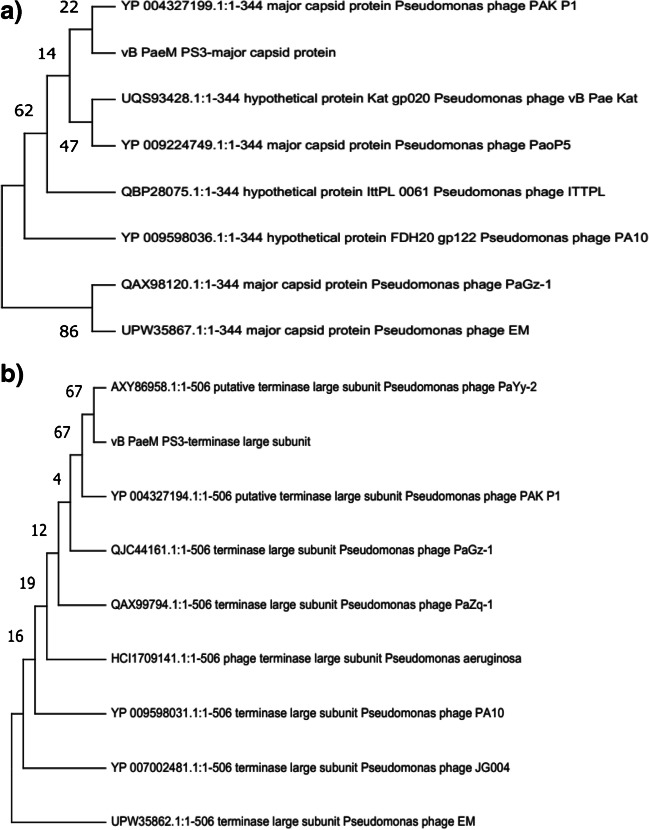
Phylogenetic trees of vB_PaeM_PS3 based on the amino acid sequences of major capsid protein (**a**) and terminase large subunit (**b**). Phylogenetic trees were constructed using MEGA-X using neighbor-joining method

The genome of vB_PaeM_PS3 comprises 93,922 bp of dsDNA with 49.39% G + C content. The isolated phage was found to be a member of the *Myoviridae* family and the genus *Pakpunavirus*. As shown in Table 2 and Fig. 6a, a total of 171 ORFs were identified, of which 27 were assigned as functional proteins whereas hypothetical proteins were encoded by 144 ORFs. The functional proteins were divided into four major classes; structure proteins (ORF 17, ORF 20, ORF 31, ORF 35, ORF 39, and ORF 41); DNA metabolism, repair and replication which involves components that help in viral replication and enzymes responsible for modification of infected cell surface polysaccharides (ORF 51, ORF 61, ORF 64, ORF 66, ORF 67, ORF 68, ORF 75, ORF 86, ORF 88, ORF 90, ORF 91, ORF 157, ORF 159, ORF 161, ORF 165, ORF 170, and ORF 171); packaging and assembly proteins (ORF 15) and finally host cell lysis (ORF 10, ORF 42, ORF 167 and ORF 168). Interestingly, a cluster of 14 tRNA genes was predicted in the phage vB_PaeM_PS3 genome and listed in Supplementary Table S1. Of note that neither lysogenic genes nor host related sequences were identified in vB_PaeM_PS3 genome, confirming the lytic nature of vB_PaeM_PS3. In addition, the genes related to *P. aeruginosa* antibiotic resistance as well as toxins and virulence proteins production were absent in vB_PaeM_PS3 genome. Phylogenetic analysis based on the overall similarity with completely sequenced phage genomes in database shows that vB_PaeM_PS3 shares greatest nucleotide similarity with *Pseudomonas* phage vB_PaeM_SCUT-S2 (GenBank Acc. No MK340761.1), *Pseudomonas* phage vB_PaM_EPA1 (GenBank Acc. No MN013356.1), *Pseudomonas* phage PaYy-2 (GenBank Acc. No MH725810.1) and *Pseudomonas* phage SRT6 (GenBank Acc. No MH370478.1) representing percent identities of 96%, 95.2%, 95%, and 94.9%, respectively (Table 3, Fig. 6b, c and Supplementary Fig. S1). Moreover, neighbor-joining (NJ) phylogenetic trees were constructed for the phage major capsid protein and terminase large subunit to better illustrate the evolutionary relationships of vB_PaeM_PS3 with other bacteriophages. As shown in Fig. 7a and b, vB_PaeM_PS3 phage displayed close relation to other *Caudovirales* related *pseudomonas* phages, specifically, *Pseudomonas* phage PAK P1 and *Pseudomonas* phage PaYy-2, respectively. These results support and are in accordance with the whole genome phylogeny.

**Table 2:** Predicted ORFs found in vB_PaeM_PS3 phage

Coding Sequence	Start…..End	GC (%)	Protein length	MW (KDa)	Gene name	Putative function	Amino acid sequence identity/similarity to best homologs	BLAST score (EValue)	Accession no
ORF1	180……392	49.77%	71	7.48	Hypothetical protein	Unknown function	Hypothetical protein IttPL_0040 [Pseudomonas phage ITTPL]	9e-41	QBP28054.1
ORF2	389……649	47.13%	87	9.66	Hypothetical protein	Unknown function	Hypothetical protein [Pseudomonas phage PaGz-1]	2e-53	QAX98104.1
ORF3	662……1321	45.45%	220	25.39	Hypothetical protein	Unknown function	Hypothetical protein X831_gp162 [Pseudomonas phage PAK_P2]	9e-163	YP_008857202.1
ORF4	1323……1670	49.43%	116	13.1	Hypothetical protein	Unknown function	Hypothetical protein PAK_P400162 [Pseudomonas phage PAK_P4]	1e-78	YP_008859373.1
ORF5	1651……1929	50.54%	93	10.63	Hypothetical protein	Unknown function	Hypothetical protein PAK_P400163 [Pseudomonas phage PAK_P4]	2e-49	YP_008859374.1
ORF6	1926……2204	48.75%	93	10.49	Hypothetical protein	Unknown function	Hypothetical protein PAK_P400164 [Pseudomonas phage PAK_P4]	3e-61	YP_008859375.1
ORF7	2239……2424	54.30%	62	7.12	Hypothetical protein	Unknown function	Hypothetical protein [Pseudomonas aeruginosa]	8e-31	WP_016064770.1
ORF8	2425……2718	54.42%	98	11.41	Hypothetical protein	Unknown function	Hypothetical protein BN405_2-10_Ab1_orf_38 [Pseudomonas phage vB_PaeM_C2-10_Ab1]	2e-66	YP_007236859.1
ORF9	2715……2900	46.77%	62	7.16	Hypothetical protein	Unknown function	Hypothetical protein PJG4_041 [Pseudomonas phage JG004]	3e-35	YP_007002487.1
ORF10	2961……3509	48.27%	183	20.4	putative protease subunit [Pseudomonas phage JG004]	Protein degradation	putative protease subunit [Pseudomonas phage JG004]	8e-110	YP_007002486.1
ORF11	3557……3913	50.42%	119	13.01	Hypothetical protein	Unknown function	Hypothetical protein PJG4_043 [Pseudomonas phage JG004]	2e-63	YP_007002485.1
ORF12	3910……4377	47.01%	156	18.07	Hypothetical protein	Unknown function	Hypothetical protein PJG4_044 [Pseudomonas phage JG004]	4e-98	YP_007002484.1
ORF13	4852……5040	47.09%	63	6.93	Hypothetical protein	Unknown function	Hypothetical protein PaP1_gp044 [Pseudomonas phage PaP1]	8e-39	YP_007236455.1
ORF14	5183……5506	44.14%	108	12.03	Hypothetical protein	Unknown function	Hypothetical protein PAK_P100177 [Pseudomonas phage PAK_P1]	1e-70	YP_004327192.1
ORF15	8877……10,397	47.53%	507	57.09	putative terminase large subunit [Pseudomonas phage PAK_P1]	Packaging process and phage assembly	putative terminase large subunit [Pseudomonas phage PAK_P1]	0.0	YP_004327194.1
ORF16	10,410……11,849	47.29%	480	54.33	Hypothetical protein	Unknown function	Hypothetical protein BN405_2-10_Ab1_orf_48 [Pseudomonas phage vB_PaeM_C2-10_Ab1]	0.0	YP_007236869.1
ORF17	11,859……12,329	49.47%	157	17.16	putative capsid and scaffold [Pseudomonas phage vB_PaeM_C2-10_Ab02]	Phage structural assembly (Head morphogenesis)	putative capsid and scaffold [Pseudomonas phage vB_PaeM_C2-10_Ab02]	2e-98	YP_009623460.1
ORF18	12,326……13,243	48.37%	306	33.07	Hypothetical protein	Unknown function	Hypothetical protein PaYy2_161 [Pseudomonas phage PaYy-2]	0.0	AXY86955.1
ORF19	13,271……13,681	50.12%	137	14.85	Hypothetical protein	Unknown function	Hypothetical protein PJG4_063 [Pseudomonas phage JG004]	2e-93	YP_007002477.1
ORF20	13,725……14,759	53.24%	345	39.38	major capsid protein [Pseudomonas phage PAK_P1]	Phage structural assembly (Head morphogenesis)	major capsid protein [Pseudomonas phage PAK_P1]	0.0	YP_004327199.1
ORF21	14,810……15,286	48.01%	159	18.16	Hypothetical protein	Unknown function	Hypothetical protein PaoP5_059 [Pseudomonas phage PaoP5]	1e-112	YP_009224750.1
ORF22	15,258……15,737	47.08%	160	18.22	Hypothetical protein	Unknown function	Hypothetical protein BN405_2-10_Ab1_orf_54 [Pseudomonas phage vB_PaeM_C2-10_Ab1]	5e-115	YP_007236875.1
ORF23	15,737……16,117	46.19%	127	14.35	Hypothetical protein	Unknown function	Hypothetical protein [Pseudomonas phage phipa10]	1e-80	UGL61562.1
ORF24	16,114……16,677	44.50%	188	21.23	Hypothetical protein	Unknown function	Hypothetical protein BN405_2-10_Ab1_orf_56 [Pseudomonas phage vB_PaeM_C2-10_Ab1]	2e-138	YP_007236877.1
ORF25	16,690……17,976	51.20%	429	46.31	Hypothetical protein	Unknown function	Hypothetical protein Kat_gp015 [Pseudomonas phage vB_Pae_Kat]	0.0	UQS93423.1
ORF26	18,007……18,531	50.10%	167	18.97	Hypothetical protein	Unknown function	Hypothetical protein BN405_2-10_Ab1_orf_59 [Pseudomonas phage vB_PaeM_C2-10_Ab1]	5e-118	YP_007236880.1
ORF27	18,606……19,106	49.90%	175	18.23	Hypothetical protein	Unknown function	Hypothetical protein PAK_P100012 [Pseudomonas phage PAK_P1]	3e-124	YP_004327205.1
ORF28	19,106……19,585	48.13%	160	17.7	Hypothetical protein	Unknown function	Hypothetical protein PJG4_073 [Pseudomonas phage JG004]	1e-112	YP_007002467.1
ORF29	19,599……19,970	47.58%	124	13.62	Hypothetical protein	Unknown function	Hypothetical protein PJG4_074 [Pseudomonas phage JG004]	1e-83	YP_007002466.1
ORF30	20,078……20,230	44.44%	51	5.58	Hypothetical protein	Unknown function	Hypothetical protein PaP1_gp161 [Pseudomonas phage PaP1]	7e-26	YP_009047070.1
ORF31	20,227……22,593	50.99%	789	85.95	putative tape measure protein [Pseudomonas phage YS35]	Tail and base plate structural component and assembly	putative tape measure protein [Pseudomonas phage YS35]	0.0	ATI16080.1
ORF32	22,590……23,351	47.24%	254	28.56	Hypothetical protein	Unknown function	Hypothetical protein Kat_gp008 [Pseudomonas phage vB_Pae_Kat]	0.0	UQS93416.1
ORF33	23,357……23,713	46.70%	119	13.99	Hypothetical protein	Unknown function	Hypothetical protein Kat_gp007 [Pseudomonas phage vB_Pae_Kat]	1e-81	UQS93415.1
ORF34	23,710……24,627	45.64%	306	33.77	Hypothetical protein	Unknown function	Hypothetical protein X831_gp020 [Pseudomonas phage PAK_P2]	0.0	YP_008857060.1
ORF35	24,624……25,364	48.72%	246	26.69	baseplate protein [Pseudomonas phage PaP1]	Tail and base plate structural component and assembly	baseplate protein [Pseudomonas phage PaP1]	0.0	YP_007236476.1
ORF36	25,375……25,746	45.97%	124	14.18	Hypothetical protein	Unknown function	Hypothetical protein BN405_2-10_Ab1_orf_68 [Pseudomonas phage vB_PaeM_C2-10_Ab1]	7e-85	YP_007236889.1
ORF37	25,748……27,211	49.11%	488	52.42	Hypothetical protein	Unknown function	Hypothetical protein Kat_gp003 [Pseudomonas phage vB_Pae_Kat]	0.0	UQS93411.1
ORF38	27,230……27,961	49.73%	244	26.69	Hypothetical protein	Unknown function	Hypothetical protein PJG4_083 [Pseudomonas phage JG004]	3e-166	YP_007002457.1
ORF39	27,972……30,029	51.55%	686	71.77	tail fiber protein [Pseudomonas phage PaoP5]	Phage assembly (Tail morphogenesis)	tail fiber protein [Pseudomonas phage PaoP5]	0.0	YP_009224767.1
ORF40	30,073……30,447	45.87%	125	14.54	Hypothetical protein	Unknown function	Hypothetical protein PJG4_085 [Pseudomonas phage JG004]	1e-76	YP_007002455.1
ORF41	30,461……31,960	51.53%	500	53	putative tail fiber protein [Pseudomonas phage C11]	Phage assembly (Tail morphogenesis)	putative tail fiber protein [Pseudomonas phage C11]	0.0	YP_009186965.1
ORF42	31,977……32,537	47.24%	187	20.93	endolysin [Pseudomonas phage vB_Pae_Kat]	Cell lysis	endolysin [Pseudomonas phage vB_Pae_Kat]	1e-134	UQS93578.1
ORF43	32,555……32,794	47.50%	80	8.46	Hypothetical protein	Unknown function	Hypothetical protein BN405_2-10_Ab1_orf_75 [Pseudomonas phage vB_PaeM_C2-10_Ab1]	7e-48	YP_007236896.1
ORF44	33,085……33,366	48.58%	94	10.54	Hypothetical protein	Unknown function	Hypothetical protein [Pseudomonas phage PaGz-1]	2e-59	QAX98142.1
ORF45	33,356……33,661	41.18%	102	11.47	Hypothetical protein	Unknown function	Hypothetical protein PJG4_091 [Pseudomonas phage JG004]	8e-65	YP_007002449.1
ORF46	33,697……34,011	46.67%	105	12.36	Hypothetical protein	Unknown function	Hypothetical protein X831_gp033 [Pseudomonas phage PAK_P2]	2e-56	YP_008857073.1
ORF47	34,049……34,360	44.87%	104	11.55	Hypothetical protein	Unknown function	Hypothetical protein PaoP5_084 [Pseudomonas phage PaoP5]	5e-58	YP_009224775.1
ORF48	34,372……34,692	45.48%	107	12.13	Hypothetical protein	Unknown function	Hypothetical protein PaoP5_085 [Pseudomonas phage PaoP5]	1e-72	YP_009224776.1
ORF49	34,694……35,506	51.54%	271	30.44	Hypothetical protein	Unknown function	Hypothetical protein [Pseudomonas phage PaZq-1]	0.0	QJC44187.1
ORF50	35,499……35,663	47.88%	55	6.02	Hypothetical protein	Unknown function	Hypothetical protein PaoP5_088 [Pseudomonas phage PaoP5]	7e-28	YP_009224779.1
ORF51	35,666……36,808	49.61%	381	42.76	putative RNA ligase [Pseudomonas phage PaoP5]	Repair, splicing and editing pathways of broken RNAs (RNA repair)	putative RNA ligase [Pseudomonas phage PaoP5]	0.0	YP_009224780.1
ORF52	36,840……37,073	47.44%	78	8.64	Hypothetical protein	Unknown function	Hypothetical protein PJG4_097 [Pseudomonas phage JG004]	1e-35	YP_007002443.1
ORF53	37,111……37,467	47.34%	119	13.17	Hypothetical protein	Unknown function	Hypothetical protein FDH21_gp131 [Pseudomonas phage Zigelbrucke]	9e-70	YP_009598139.1
ORF54	37,801……37,998	52.53%	66	7.47	Hypothetical protein	Unknown function	Hypothetical protein BN405_2-10_Ab1_orf_84 [Pseudomonas phage vB_PaeM_C2-10_Ab1]	1e-37	YP_007236905.1
ORF55	38,001……38,486	51.44%	162	18.92	Hypothetical protein	Unknown function	Hypothetical protein S2_091 [Pseudomonas phage vB_PaeM_SCUT-S2]	7e-115	QAU05363.1
ORF56	38,519……38,773	52.16%	85	9.51	Hypothetical protein	Unknown function	Hypothetical protein Kat_gp156 [Pseudomonas phage vB_Pae_Kat]	1e-38	UQS93564.1
ORF57	38,775……39,164	45.90%	130	14.85	Hypothetical protein	Unknown function	Hypothetical protein [Pseudomonas phage PaGz-1]	1e-91	QAX98151.1
ORF58	39,161……39,802	50.47%	214	23.48	Hypothetical protein	Unknown function	Hypothetical protein Kat_gp154 [Pseudomonas phage vB_Pae_Kat]	5e-153	UQS93562.1
ORF59	39,789……39,953	44.24%	55	6.39	Hypothetical protein	Unknown function	Hypothetical protein [Pseudomonas phage PaGz-1]	2e-30	QAX98153.1
ORF60	39,956……40,258	47.19%	101	11.78	Hypothetical protein	Unknown function	Hypothetical protein [Pseudomonas phage PaGz-1]	1e-67	QAX98154.1
ORF61	40,259……40,681	48.94%	141	16.51	anaerobic NTP reductase large subunit [Pseudomonas phage JHP]	DNA replication and repair	anaerobic NTP reductase large subunit [Pseudomonas phage JHP]	3e-101	QBJ04655.1
ORF62	40,690……40,818	41.86%	43	5.1	Hypothetical protein	Unknown function	Hypothetical protein PAK_P100050 [Pseudomonas phage PAK_P1]	7e-08	YP_008869167.1
ORF63	40,805……40,996	51.04%	64	7.12	Hypothetical protein	Unknown function	Hypothetical protein PaP1_gp087 [Pseudomonas phage PaP1]	9e-37	YP_007236498.1
ORF64	41,006……41,251	52.44%	82	9.21	putative DNA primase/helicase [Pseudomonas phage vB_PaeM_SCUT-S2]	DNA replication	putative DNA primase/helicase [Pseudomonas phage vB_PaeM_SCUT-S2]	2e-53	QAU05373.1
ORF65	41,248……41,433	51.08%	62	7.17	Hypothetical protein	Unknown function	Hypothetical protein [Pseudomonas phage PaGz-1]	5e-38	QAX98158.1
ORF66	41,487……43,349	51.26%	621	70.57	putative DNA primase/helicase [Pseudomonas phage PAK_P1]	DNA replication	putative DNA primase/helicase [Pseudomonas phage PAK_P1]	0.0	YP_004327242.1
ORF67	43,410……45,419	50.05%	670	77.47	putative DNA polymerase [Pseudomonas phage PAK_P1]	DNA replication	putative DNA polymerase [Pseudomonas phage PAK_P1]	0.0	YP_008869170.1
ORF68	45,704……46,393	51.30%	230	25.44	DNA polymerase A family protein [Pseudomonas phage K8]	DNA replication	DNA polymerase A family protein [Pseudomonas phage K8]	4e-152	YP_009200042.1
ORF69	46,483……46,881	51.63%	133	14.21	Hypothetical protein	Unknown function	Hypothetical protein [Pseudomonas phage PaZq-1]	1e-87	QAX99843.1
ORF70	46,911……47,078	46.43%	56	6.15	Hypothetical protein	Unknown function	Hypothetical protein PaP1_gp092 [Pseudomonas phage PaP1]	2e-31	YP_007236503.1
ORF71	47,080……47,796	50.49%	239	26.63	Hypothetical protein	Unknown function	Hypothetical protein PJG4_113 [Pseudomonas phage JG004]	3e-142	YP_007002427.1
ORF72	47,898……48,902	51.74%	335	37.15	Hypothetical protein	Unknown function	Hypothetical protein [Pseudomonas phage PaZq-1]	0.0	QAX99846.1
ORF73	48,965……49,198	42.31%	78	8.6	Hypothetical protein	Unknown function	Hypothetical protein PAK_P100060 [Pseudomonas phage PAK_P1]	3e-28	YP_004327249.1
ORF74	49,208……49,429	44.59%	74	8.25	Hypothetical protein	Unknown function	Hypothetical protein PAK_P100061 [Pseudomonas phage PAK_P1]	3e-45	YP_004327250.1
ORF75	49,471……50,523	50.90%	351	40.03	Putative exodeoxyribonuclease [Pseudomonas phage vB_PaeM_C2-10_Ab1]	DNA replication, modification and regulation	Putative exodeoxyribonuclease [Pseudomonas phage vB_PaeM_C2-10_Ab1]	0.0	YP_007236926.1
ORF76	50,520……51,083	50.53%	188	21.51	Hypothetical protein	Unknown function	Hypothetical protein PaoP5_115 [Pseudomonas phage PaoP5]	6e-135	YP_009224805.1
ORF77	51,080……51,466	48.32%	129	15.05	Hypothetical protein	Unknown function	Hypothetical protein PaoP5_116 [Pseudomonas phage PaoP5]	4e-89	YP_009224806.1
ORF78	51,463……51,693	47.19%	77	8.61	Hypothetical protein	Unknown function	Hypothetical protein [Pseudomonas phage PaZq-1]	5e-46	QJC44194.1
ORF79	51,690……52,127	46.35%	146	16.46	Hypothetical protein	Unknown function	Hypothetical protein PaoP5_118 [Pseudomonas phage PaoP5]	2e-102	YP_009224808.1
ORF80	52,124……52,294	46.20%	57	6.51	Hypothetical protein	Unknown function	Hypothetical protein PaoP5_119 [Pseudomonas phage PaoP5]	8e-33	YP_009224809.1
ORF81	52,291……53,070	51.03%	260	29.34	Hypothetical protein	Unknown function	Hypothetical protein PJG4_124 [Pseudomonas phage JG004]	0.0	YP_007002416.1
ORF82	53,067……53,249	45.36%	61	6.9	Hypothetical protein	Unknown function	Hypothetical protein X831_gp070 [Pseudomonas phage PAK_P2]	7e-35	YP_008857110.1
ORF83	53,261……53,470	45.71%	70	7.5	Hypothetical protein	Unknown function	Hypothetical protein [Pseudomonas phage PhL_UNISO_PA-DSM_ph0034]	9e-31	QYC95157.1
ORF84	53,489……53,824	52.38%	112	12.44	Hypothetical protein	Unknown function	Hypothetical protein PAK_P100072 [Pseudomonas phage PAK_P1]	1e-73	YP_004327259.1
ORF85	53,828……54,040	47.42%	71	7.72	Hypothetical protein	Unknown function	Hypothetical protein PJG4_128 [Pseudomonas phage JG004]	1e-40	YP_007002412.1
ORF86	54,033……54,989	48.38%	319	35.56	3'-phosphatase, 5'-polynucleotide kinase [Pseudomonas phage PaZq-1]	DNA metabolism and replication	3'-phosphatase, 5'-polynucleotide kinase [Pseudomonas phage PaZq-1]	0.0	QAX99857.1
ORF87	55,046……55,138	44.09%	31	3.55	Hypothetical protein	Unknown function	Hypothetical protein Kat_gp125 [Pseudomonas phage vB_Pae_Kat]	5e-12	UQS93533.1
ORF88	55,193……55,906	48.88%	238	27.17	FAD-dependent thymidylate synthase [Pseudomonas aeruginosa]	Nucleotide metabolism and DNA replication	FAD-dependent thymidylate synthase [Pseudomonas aeruginosa]	3e-131	HCI1709201.1
ORF89	56,160……56,504	48.99%	115	13.19	Hypothetical protein	Unknown function	Hypothetical protein Y35_GM000048 [Pseudomonas phage YS35]	4e-80	ATI16021.1
ORF90	56,521……57,567	47.76%	349	40.34	ribonucleotide-diphosphate reductase beta subunit [Pseudomonas phage vB_PaeM_C2-10_Ab1]	DNA replication and repair	ribonucleotide-diphosphate reductase beta subunit [Pseudomonas phage vB_PaeM_C2-10_Ab1]	0.0	YP_007236938.1
ORF91	57,560……59,305	51.32%	582	66.64	ribonucleoside-diphosphate reductase alpha chain [Pseudomonas phage PaGz-1]	DNA replication and repair	ribonucleoside-diphosphate reductase alpha chain [Pseudomonas phage PaGz-1]	0.0	QAX98182.1
ORF92	59,380……59,526	50.34%	49	5.62	Hypothetical protein	Unknown function	Hypothetical protein [Pseudomonas phage PaGz-1]	3e-26	QAX98183.1
ORF93	59,523……59,759	51.05%	79	9.41	Hypothetical protein	Unknown function	Hypothetical protein [Pseudomonas phage PaGz-1]	2e-49	QAX98184.1
ORF94	59,759……59,980	45.05%	74	8.38	Hypothetical protein	Unknown function	Hypothetical protein [Pseudomonas phage PaGz-1]	8e-47	QAX98185.1
ORF95	59,993……60,229	46.41%	79	9.07	Hypothetical protein	Unknown function	Hypothetical protein [Pseudomonas phage PaGz-1]	1e-48	QAX98186.1
ORF96	60,229……60,498	51.48%	90	10.16	Hypothetical protein	Unknown function	Hypothetical protein [Pseudomonas phage PaGz-1]	2e-60	QAX98187.1
ORF97	60,500……60,814	50.79%	105	12.03	Hypothetical protein	Unknown function	Hypothetical protein [Pseudomonas phage PaGz-1]	2e-57	QAX98188.1
ORF98	60,804……61,004	47.76%	67	7.56	Hypothetical protein	Unknown function	Hypothetical protein [Pseudomonas phage PaGz-1]	4e-41	QAX98189.1
ORF99	61,049……61,294	41.06%	82	9.36	Hypothetical protein	Unknown function	Hypothetical protein [Pseudomonas phage PaGz-1]	7e-51	QAX98190.1
ORF100	61,307……61,810	48.02%	168	18.36	Hypothetical protein	Unknown function	Hypothetical protein [Pseudomonas phage PaGz-1]	4e-119	QAX98191.1
ORF101	61,820……62,014	49.23%	65	7.3	Hypothetical protein	Unknown function	Hypothetical protein PAK_P100089 [Pseudomonas phage PAK_P1]	2e-38	YP_004327273.1
ORF102	62,016……62,246	50.22%	77	8.69	Hypothetical protein	Unknown function	Hypothetical protein [Pseudomonas phage PaGz-1]	6e-48	QAX98193.1
ORF103	62,319……62,519	51.24%	67	8.18	Hypothetical protein	Unknown function	Hypothetical protein PaP1_gp124 [Pseudomonas phage PaP1]	1e-39	YP_007236535.1
ORF104	62,677……63,663	49.24%	329	37.56	Hypothetical protein	Unknown function	Hypothetical protein [Pseudomonas phage PaGz-1]	0.0	QAX98195.1
ORF105	63,886……64,059	44.25%	58	6.92	Hypothetical protein	Unknown function	Hypothetical protein PAK_P100093 [Pseudomonas phage PAK_P1]	4e-33	YP_008869183.1
ORF106	64,689……65,165	50.52%	159	18.15	Hypothetical protein	Unknown function	Hypothetical protein [Pseudomonas phage PhL_UNISO_PA-DSM_ph0034]	2e-113	QYC95184.1
ORF107	65,240……65,506	50.56%	89	10.3	Hypothetical protein	Unknown function	Hypothetical protein BI047_gp158 [Pseudomonas phage phiMK]	7e-58	YP_009291099.1
ORF108	65,519……65,659	48.94%	47	5.31	Hypothetical protein	Unknown function	Hypothetical protein Kat_gp104 [Pseudomonas phage vB_Pae_Kat]	8e-26	UQS93512.1
ORF109	65,662……65,949	51.74%	96	10.58	Hypothetical protein	Unknown function	Hypothetical protein PaP1_gp129 [Pseudomonas phage PaP1]	5e-61	YP_007236540.1
ORF110	65,961……66,194	52.56%	78	8.42	Hypothetical protein	Unknown function	Hypothetical protein K8_149 [Pseudomonas phage K8]	8e-48	YP_009200085.1
ORF111	66,265……66,393	51.16%	43	4.84	Hypothetical protein	Unknown function	Hypothetical protein PAK_P100099 [Pseudomonas phage PAK_P1]	2e-20	YP_004327282.1
ORF112	66,393……66,701	51.13%	103	11.41	Hypothetical protein	Unknown function	Hypothetical protein X831_gp098 [Pseudomonas phage PAK_P2]	9e-69	YP_008857138.1
ORF113	66,775……66,921	52.38%	49	5.58	Hypothetical protein	Unknown function	Hypothetical protein PaP1_gp133 [Pseudomonas phage PaP1]	3e-11	YP_007236544.1
ORF114	67,158……67,541	52.60%	128	15.09	Hypothetical protein	Unknown function	Hypothetical protein PJG4_153 [Pseudomonas phage JG004]	2e-87	YP_007002545.1
ORF115	67,617……68,288	52.53%	224	24.41	Hypothetical protein	Unknown function	Hypothetical protein [Pseudomonas phage PaGz-1]	1e-166	QAX98206.1
ORF116	68,293……68,631	51.33%	113	12.58	Hypothetical protein	Unknown function	Hypothetical protein X831_gp104 [Pseudomonas phage PAK_P2]	2e-79	YP_008857144.1
ORF117	68,631……68,999	50.41%	123	13.49	Hypothetical protein	Unknown function	Hypothetical protein [Pseudomonas phage PaGz-1]	6e-85	QAX98208.1
ORF118	68,996……69,268	52.01%	91	10.41	Hypothetical protein	Unknown function	Hypothetical protein FDH21_gp066 [Pseudomonas phage Zigelbrucke]	7e-59	YP_009598204.1
ORF119	69,265……69,459	50.26%	65	6.95	Hypothetical protein	Unknown function	Hypothetical protein PJG4_158 [Pseudomonas phage JG004]	2e-38	YP_007002540.1
ORF120	69,477……69,773	45.79%	99	11.43	Hypothetical protein	Unknown function	Hypothetical protein [Pseudomonas phage PaGz-1]	1e-65	QAX98210.1
ORF121	69,770……69,994	54.67%	75	8.38	Hypothetical protein	Unknown function	Hypothetical protein AU075_gp065 [Pseudomonas phage C11]	3e-38	YP_009187045.1
ORF122	70,027……70,293	53.93%	89	9.96	Hypothetical protein	Unknown function	Hypothetical protein PAK_P100112 [Pseudomonas phage PAK_P1]	1e-57	YP_004327292.1
ORF123	70,290……70,682	51.40%	131	15.07	Hypothetical protein	Unknown function	Hypothetical protein PaP1_gp142 [Pseudomonas phage PaP1]	1e-91	YP_007236553.1
ORF124	70,797……71,381	48.03%	195	21.74	Hypothetical protein	Unknown function	Hypothetical protein [Pseudomonas phage PaGz-1]	1e-128	QAX98214.1
ORF125	71,453……71,698	49.59%	82	9.14	Hypothetical protein	Unknown function	Hypothetical protein PaP1_gp144 [Pseudomonas phage PaP1]	4e-53	YP_007236555.1
ORF126	71,710……72,039	48.48%	110	12.5	Hypothetical protein	Unknown function	Hypothetical protein [Pseudomonas phage PaGz-1]	3e-75	QAX98216.1
ORF127	72,076……72,573	52.61%	166	18.15	Hypothetical protein	Unknown function	Hypothetical protein [Pseudomonas phage PaGz-1]	3e-119	QAX98217.1
ORF128	72,657……73,172	52.33%	172	19.62	Hypothetical protein	Unknown function	Hypothetical protein [Pseudomonas phage PaGz-1]	5e-123	QAX98055.1
ORF129	73,248……73,775	56.25%	176	19.5	Hypothetical protein	Unknown function	Hypothetical protein BI047_gp010 [Pseudomonas phage phiMK]	1e-111	YP_009291077.1
ORF130	73,844……73,960	48.72%	39	4.43	Hypothetical protein	Unknown function	Hypothetical protein PaSzw1_62 [Pseudomonas phage PaSzW-1]	1e-18	QAY01625.1
ORF131	74,066……74,260	53.85%	65	7.6	Hypothetical protein	Unknown function	MULTISPECIES: DUF551 domain-containing protein [Pseudomonas]	4e-39	WP_088172792.1
ORF132	74,298……74,462	45.45%	55	6.21	Hypothetical protein	Unknown function	Hypothetical protein BI047_gp007 [Pseudomonas phage phiMK]	1e-30	YP_009291074.1
ORF133	74,543……74,689	51.70%	49	5.58	Hypothetical protein	Unknown function	Hypothetical protein BI047_gp006 [Pseudomonas phage phiMK]	4e-26	YP_009291073.1
ORF134	74,751……75,212	51.52%	154	17.15	Hypothetical protein	Unknown function	Hypothetical protein BI047_gp005 [Pseudomonas phage phiMK]	3e-110	YP_009291072.1
ORF135	75,229……75,516	55.90%	96	10.92	Hypothetical protein	Unknown function	Hypothetical protein Kat_gp080 [Pseudomonas phage vB_Pae_Kat]	9e-62	UQS93488.1
ORF136	75,590……75,820	53.68%	77	8.19	Hypothetical protein	Unknown function	Hypothetical protein FBPa2_0081 [Pseudomonas phage vB_PaeM_FBPa2]	2e-39	UVN13020.1
ORF137	75,894……76,088	50.77%	65	7.1	Hypothetical protein	Unknown function	Hypothetical protein [Pseudomonas phage PhL_UNISO_PA-DSM_ph0034]	3e-38	QYC95212.1
ORF138	76,138……76,308	57.89%	57	6.57	Hypothetical protein	Unknown function	Hypothetical protein FDH21_gp050 [Pseudomonas phage Zigelbrucke]	5e-24	YP_009598220.1
ORF139	76,471……76,629	57.86%	53	6.18	Hypothetical protein	Unknown function	Hypothetical protein PAK_P100128 [Pseudomonas phage PAK_P1]	2e-30	YP_008869200.1
ORF140	77,422……78,015	48.82%	198	22.68	Hypothetical protein	Unknown function	Hypothetical protein [Pseudomonas phage vB_PaeM_C2-10_Ab08]	1e-143	CEF89317.1
ORF141	78,528……78,773	48.37%	82	9.75	Hypothetical protein	Unknown function	Hypothetical protein BN405_2-10_Ab1_orf_02 [Pseudomonas phage vB_PaeM_C2-10_Ab1]	1e-53	YP_007236823.1
ORF142	78,773……79,177	51.11%	135	15.27	Hypothetical protein	Unknown function	Hypothetical protein X831_gp128 [Pseudomonas phage PAK_P2]	4e-92	YP_008857168.1
ORF143	79,167…….79583	46.28%	139	16.01	Hypothetical protein	Unknown function	Hypothetical protein FDH21_gp004 [Pseudomonas phage Zigelbrucke]	8e-98	YP_009598056.1
ORF144	79,606…….80292	50.51%	229	27.13	Hypothetical protein	Unknown function	Hypothetical protein PaP1_gp005 [Pseudomonas phage PaP1]	2e-153	YP_007236416.1
ORF145	80,295……80,576	50.35%	94	10.57	Hypothetical protein	Unknown function	Hypothetical protein BN405_2-10_Ab1_orf_06 [Pseudomonas phage vB_PaeM_C2-10_Ab1]	1e-41	YP_007236827.1
ORF146	80,564……80,875	46.47%	104	11.99	Hypothetical protein	Unknown function	Hypothetical protein FDH21_gp006 [Pseudomonas phage Zigelbrucke]	1e-66	YP_009598058.1
ORF147	80,875……81,237	50.14%	121	13.85	Hypothetical protein	Unknown function	Hypothetical protein PAK_P400132 [Pseudomonas phage PAK_P4]	2e-83	YP_008859343.1
ORF148	81,294……81,509	48.15%	72	8.33	Hypothetical protein	Unknown function	Hypothetical protein PAK_P100139c [Pseudomonas phage PAK_P1]	9e-44	YP_004327153.1
ORF149	81,506……81,847	50.88%	114	12.65	Hypothetical protein	Unknown function	Hypothetical protein PJG4_011 [Pseudomonas phage JG004]	9e-78	YP_007003112.1
ORF150	81,849……82,352	49.60%	168	19.31	Hypothetical protein	Unknown function	Hypothetical protein BN405_2-10_Ab1_orf_12 [Pseudomonas phage vB_PaeM_C2-10_Ab1]	3e-121	YP_007236833.1
ORF151	82,339……82,716	49.47%	126	14.99	Hypothetical protein	Unknown function	Hypothetical protein Henu5_gp69 [Pseudomonas phage Henu5]	2e-87	QAU05099.1
ORF152	82,736……83,317	51.72%	194	21.62	Hypothetical protein	Unknown function	Hypothetical protein Kat_gp060 [Pseudomonas phage vB_Pae_Kat]	8e-130	UQS93468.1
ORF153	83,314……83,625	44.87%	104	12.08	Hypothetical protein	Unknown function	Hypothetical protein Kat_gp059 [Pseudomonas phage vB_Pae_Kat]	3e-69	UQS93467.1
ORF154	83,627……83,776	48.67%	50	5.68	Hypothetical protein	Unknown function	Hypothetical protein PaP1_gp015 [Pseudomonas phage PaP1]	1e-08	YP_007236426.1
ORF155	83,788……84,267	52.71%	160	17.77	Hypothetical protein	Unknown function	Hypothetical protein Kat_gp057 [Pseudomonas phage vB_Pae_Kat]	2e-113	UQS93465.1
ORF156	84,264……84,461	39.39%	66	7.85	Hypothetical protein	Unknown function	Hypothetical protein [Pseudomonas phage PaGz-1]	4e-41	QAX98087.1
ORF157	84,472……86,160	50.98%	563	62.92	nicotinamide phosphoribosyltransferase [Pseudomonas phage vB_Pae_Kat]	DNA modification	nicotinamide phosphoribosyltransferase [Pseudomonas phage vB_Pae_Kat]	0.0	UQS93463.1
ORF158	86,217……86,435	44.75%	73	8.78	Hypothetical protein	Unknown function	Hypothetical protein Kat_gp054 [Pseudomonas phage vB_Pae_Kat]	8e-43	UQS93462.1
ORF159	86,432……87,307	49.20%	292	31.71	ribose-phosphate pyrophosphokinase [Pseudomonas phage vB_Pae_Kat]	DNA metabolism and replication	ribose-phosphate pyrophosphokinase [Pseudomonas phage vB_Pae_Kat]	0.0	UQS93461.1
ORF160	87,318……87,737	52.14%	140	15.86	Hypothetical protein	Unknown function	Hypothetical protein [Pseudomonas phage PaGz-1]	1e-97	QAX98091.1
ORF161	87,748……88,665	49.24%	306	34.82	putative RNA ligase/tail attachment protein [Pseudomonas phage JG004]	DNA modification and repair	putative RNA ligase/tail attachment protein [Pseudomonas phage JG004]	0.0	YP_007002506.1
ORF162	88,677……89,084	47.55%	136	15.13	Hypothetical protein	Unknown function	Hypothetical protein [Pseudomonas phage vB_PaeM_LCK69]	9e-59	AZF89735.1
ORF163	89,081……89,356	45.65%	92	10.42	Hypothetical protein	Unknown function	Hypothetical protein PJG4_024 [Pseudomonas phage JG004]	5e-61	YP_007002504.1
ORF164	89,358……89,594	46.41%	79	8.86	Hypothetical protein	Unknown function	Hypothetical protein PJG4_025 [Pseudomonas phage JG004]	1e-48	YP_007002503.1
ORF165	89,607……90,161	47.93%	185	21.93	putative phosphoesterase [Pseudomonas phage vB_PaeM_C2-10_Ab1]	Splicing of t-RNA introns and cellular processes Or, DNA metabolism and replication	putative phosphoesterase [Pseudomonas phage vB_PaeM_C2-10_Ab1]	4e-119	YP_007236845.1
ORF166	90,161……90,592	48.61%	144	17.02	Hypothetical protein	Unknown function	Hypothetical protein PJG4_027 [Pseudomonas phage JG004]	3e-98	UQS93454.1
ORF167	90,582……91,142	49.02%	187	21.08	putative phosphohydrolase [Pseudomonas phage JG004]	Cell lysis (hydrolyzing peptidoglycan)	putative phosphohydrolase [Pseudomonas phage JG004]	5e-126	YP_007002500.1
ORF168	91,144……91,704	50.45%	187	21.48	putative cell wall hydrolase [Pseudomonas phage vB_PaeM_C2-10_Ab1]	Bacterial cell lysis	putative cell wall hydrolase [Pseudomonas phage vB_PaeM_C2-10_Ab1]	1e-137	YP_007236848.1
ORF169	91,769……92,230	48.05%	154	17.26	Hypothetical protein	Unknown function	Hypothetical protein Henu5_gp86 [Pseudomonas phage Henu5]	3e-110	QAU05115.1
ORF170	92,243……93,451	49.46%	403	46.33	putative DNA ligase [Pseudomonas phage PaoP5]	DNA replication and repair	putative DNA ligase [Pseudomonas phage PaoP5]	0.0	YP_009224726.1
ORF171	93,448……93,864	50.60%	139	15.41	putative CMP/dCMP deaminase, zinc-binding [Pseudomonas phage PAK_P1]	Nucleotide metabolism and DNA replication	putative CMP/dCMP deaminase, zinc-binding [Pseudomonas phage PAK_P1]	2e-95	YP_004327179.1

**Table 3:** Homology of phage vB_PaeM_PS3 to other phages genomes

Scientific Name	Percent Identity	Accession Length (bp)	Accession No
*Pseudomonas* phage vB_PaeM_SCUT-S2	96%	94,434	MK340761.1
*Pseudomonas* phage vB_PaM_EPA1	95.20%	91,394	MN013356.1
*Pseudomonas* phage PaYy-2	95.00%	92,348	MH725810.1
*Pseudomonas* phage SRT6	94.90%	91,364	MH370478.1
*Pseudomonas* phage PaoP5	94.50%	93,464	KU297675.1
*Pseudomonas* phage C11	94.08%	94,109	KT804923.1
*Pseudomonas* phage phiMK	94.52%	93,129	KU761955.1
*Pseudomonas* phage PhL_UNISO_PA-DSM_ph0034	96.77%	92,408	MW526259.2
*Pseudomonas* phage Zigelbrucke	96.04%	92,338	NC_041904.1
*Pseudomonas* phage PaGz-1	94.00%	93,975	MH791399.2

### *The phage vB_PaeM_PS3 exhibits a potent *in vivo* antibacterial activity against P. aeruginosa*

**Fig. 8:**
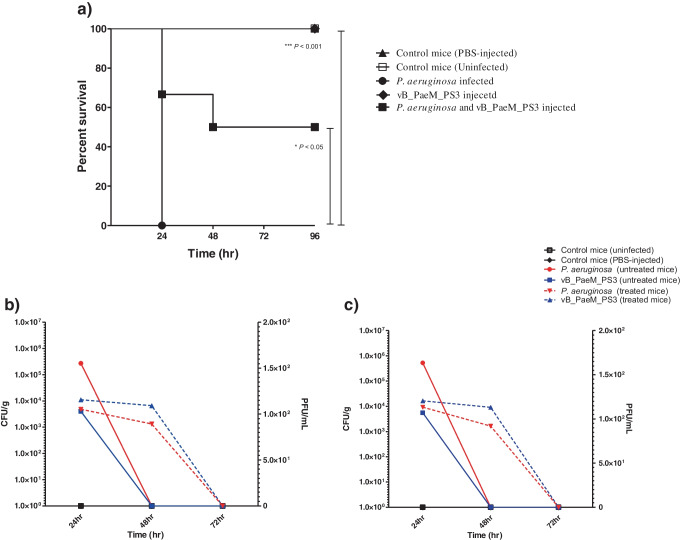
Impact of phage vB_PaeM_PS3 on *P. aeruginosa* pathogenesis in vivo. **a**) Survival rate of mice infected with *P. aeruginosa* and treated with isolated phage. Mice survival was plotted using Kaplan–Meier survival curve and statically analyzed using Log-rank test. Bacterial count and phage titer in the liver (**b**) and spleen (**c**) isolated from mice at 24, 48, and 72 h after infection. Bacterial and phage load were represented on left and right y axis, respectively. Data are presented as means ± SE of three independent experiments

The impact of vB_PaeM_PS3 on *P. aeruginosa* virulence was assessed in vivo. The mice infected with *P. aeruginosa* showed 100% mortality rate at 24 h. On contrast, the survival rate of *P. aeruginosa* infected mice was significantly improved (66.7%) following treatment with vB_PaeM_PS3 (Fig. 8a). Importantly, no death was recorded either in mice injected with vB_PaeM_PS3 alone or negative control mice and they remained healthy over the course of experiment. Additionally, both bacterial burden and vB_PaeM_PS3 titer were determined in isolated mice organs. Interestingly, treatment of *P. aeruginosa*-infected mice with vB_PaeM_PS3 effectively lowered *Pseudomonas* colonization in mice organs. The results revealed that bacterial loads in liver and spleen isolated from *P. aeruginosa* mice were markedly higher (27 × 10^4^ ± 28, 52 × 10^4^ ± 21 CFUs/g, respectively) compared to those of phage-untreated mice (4837 ± 16, 9210 ± 17 CFUs/g, respectively) (Fig. 8b, c). It is worth mentioning that the phage vB_PaeM_PS3 was completely eliminated and not detected in organs isolated from mice at 72 h post-inoculation.

## Discussion

*P. aeruginosa* is a serious nosocomial pathogen which causes a variety of health illnesses due to increased prevalence of resistant strains [54]. Therefore, phage therapy has received a significant interest as an alternative to antibiotics as well as a new strategy to alleviate antimicrobial resistance [55]. One of the major benefits of phage therapy over antibiotics is host specificity without influencing human microbial flora [56]. Consequently, isolation and characterization of new lytic phages targeting antibiotic resistant *P. aeruginosa* would be helpful to combat these infections that threaten human healthcare.

Lytic phages are more preferred for phage therapy as compared with temperate ones that could lysogenize bacterial cells. In addition, toxin genes should be absent in the genomes of phages applied for phage therapy [57]. In this context, a novel virulent phage vB_PaeM_PS3 targeting *P. aeruginosa* was isolated herein and fully characterized. The morphological features coupled with genomic analysis revealed that vB_PaeM_PS3 belonging to the family *Myoviridae* and order *Caudovirales* that represents approximately 95% of isolated phages [58]. The phage vB_PaeM_PS3 exhibited a relatively strong lytic potential on tested *P. aeruginosa* isolates. These findings were further confirmed by EOP analysis which is considered a rigorous test for evaluating phage infectivity indicated the productive infectivity of vB_PaeM_PS3 and releasing of new progeny rather than by non-productive infection or lysis from without [59]. Additionally, the isolated phage demonstrated a reasonable environmental stability and optimal growth over a broad range of temperature and pH ranges. Phage tolerance to various environmental conditions is very relevant if phage would be involved in therapeutic applications as well as during storage and production process [60]. This finding agrees with earlier studies that reported that the majority of phages survive well at pH range of 5 to 9 [61]. The vB_PaeM_PS3 phage has a latent period of 10 min and burst size of approximately 132 virions per infected cell with a higher likelihood of previously isolated phage Ps12-on-D with latent period of 10 min and an average size of 115 PFU/infected cell [62]. Phages had short latent period and large burst size are more effective and preferable for clinical use [63]. Consequently, vB_PaeM_PS3 could be considered as a good candidate for biocontrol and phage therapy.

The combination of phage and antibiotics is of great importance to avoid the development of antibiotic resistant bacterial strains [64]. Several studies have demonstrated that phages could produce synergistic antimicrobial effects when combined with conventional antibiotics [65]. Current results show a synergetic effect of vB_PaeM_PS3 when combined with traditionally used antibiotics in the treatment of *pseudomonas* infections, hence supporting promising application of isolated phage for therapy.

The most critical aspect associated with *P. aeruginosa* infections is biofilm formation [66, 67]. Fortunately, phages have been found to be highly effective in treatment of recalcitrant infections and biofilm removal [68]. The most common mechanism of phage antibiofilm activity could be due to production of different enzymes such as depolymerases and endolysins [69]. Depolymerase can specifically degrade the extracellular polysaccharides in bacterial matrix facilitating phage adsorption, penetration and lysis of bacterial cell [70]. Furthermore, endolysins can degrade bacterial peptidoglycan and assist in release of new phage progeny out of host cell [71]. Current results show that vB_PaeM_PS3 possesses a potent antibiofilm activity and therefore is an attractive biological agent for therapeutic application.

In order to consider phages for in vivo therapeutic applications, it is important to ascertain that phage genome devoid of any lysogenic, antibiotic resistance and virulence-related genes [72]. Importantly, the analysis of vB_PaeM_PS3 genome revealed the absence of both virulence-encoding and lysogeny genes indicating the true lytic nature of bacteriophage. Furthermore, phage genome analysis allowed the detection of 14 tRNAs. The presence of tRNAs is often found in myoviruses with large genomes and is common in strictly virulent or lytic phages [73, 74]. The phage-encoded tRNA genes are generally present in clusters and promote an efficient and more rapid translation rate [75]. In addition, it is thought that tRNAs are responsible for phages have short latent period and large burst size because. It has been reported that tRNAs enable phages propagation and enhance viral replication kinetics [76].

The above findings become more convincing in light of previous reports that documented the effectiveness of local and systemic application of phages in controlling infections by *P. aeruginosa* [77]. For instance, Jeon and Yong found that phage nasal inhalation effectively reduced *P. aeruginosa* count in mice lungs with pneumonia [78]. Similarly, other successful clinical trials revealed the efficacy of phage therapy against ear and burn infections caused by *P. aeruginosa* [79, 80]. Intriguingly, the phage vB_PaeM_PS3 has been found to be highly efficient in attenuation of *P. aeruginosa* virulence in mice, which further encourages its future application in phage therapy. Not only vB_PaeM_PS3 enhanced survival of *P. aeruginosa*-infected mice, it markedly reduced bacterial proliferation and colonization in tissues obtained from infected mice. It is worth mentioning that no detectable side effects or harmful signs were observed in mice upon phage treatment over the experiment course. Given the promising results of this study, it may be useful to incorporate vB_PaeM_PS3 phage in treatment of human infections with *P. aeruginosa*.

In conclusion, vB_PaeM_PS3 phage is a novel lytic phage, member of the family *Myoviridae* and the genus *Pakpunavirus.* The phage vB_PaeM_PS3 exhibited an optimum environmental stability added to its effective antimicrobial activity and the ability to disrupt bacterial biofilms. Genomic analysis revealed that phage vB_PaeM_PS3 does not contain any toxic genes or integrases and the detected tRNAs could further enhance phage protein translation that make the phage independent on the host. Furthermore, in vivo results proved the safety and efficiency of isolated phage against *P. aeruginosa* as an alternative to traditionally used antibiotics. Based on these findings, the phage vB_PaeM_PS3 fulfills all requirements of effective phages for further application as an innovative candidate against *P. aeruginosa* infections. However, further investigations about phage formulations are required to be involved in clinical trials.

### Supplementary Information

Below is the link to the electronic supplementary material.

Supplementary file1 (PDF 727 KB)

## Data Availability

The authors confirm that the data supporting the findings of this study are available within the article.

## Abbreviations

MDR: Multidrug resistant
ORFs: Open reading frames
WHO: World Health Organization
TS: Tryptone soya
TEM: Transmission electron microscopy
EOP: Efficiency of plating
PFUs: Plaque forming units
CFUs: Colony-forming units
MOI: Multiplicity of infection
OD: Optical density
BIMs: Bacteriophage insensitive mutants
FICI: Fractional inhibitory concentration index
MIC: Minimum inhibitory concentration
QUAST: Quality Assessment Tool for Genome Assemblies
RAST: Rapid Annotation Subsystems Technology
NCBI: National Center for Biotechnology Information
IP: Intraperitoneally
ICTV: International Committee Taxonomy of Viruses
NJ: Neighbor-joining
