# Supplementary material for: A novel lytic phage exhibiting a remarkable in vivo therapeutic potential and higher antibiofilm activity against Pseudomonas aeruginosa
Source: Eur J Clin Microbiol Infect Dis. 2023 Aug 23;42(10):1207–34. doi: 10.1007/s10096-023-04649-y (PMC10511388; doi:10.1007/s10096-023-04649-y)

Supplementary information

European Journal of Clinical Microbiology & Infectious Diseases

A novel lytic phage exhibiting a remarkable in vivo therapeutic potential and higher antibiofilm activity against *Pseudomonas aeruginosa*

Aliaa Abdelghafar, Amira El-Ganiny, Ghada Shaker and Momen Askoura*

Department of Microbiology and Immunology, Faculty of Pharmacy,
Zagazig University, Zagazig 44519, Egypt

* Corresponding author:

Momen Askoura: MMAskoura@pharmacy.zu.edu.eg; momenaskora@yahoo.com;
ORCiD: 0000-0002-4282-6789

Supplementary Table S1: Details of tRNAs predicted in the vB_PaeM_PS3 genome

tRNA No.	Location (bp)	Length (bp)	Amino acid	Anticodon
1	5512-5585	74	Gln	TTG
2	5892-5966	75	Arg	TCT
3	5976-6052	77	Lys	TTT
4	6327-6411	85	Leu	TAG
5	6620-6695	76	Ile	GAT
6	6705-6783	79	Asp	GTC
7	7195-7270	76	Cys	GCA
8	7281-7356	76	Asn	GTT
9	7419-7496	78	Pro	TGG
10	7667-7742	76	Gly	TCC
11	8263-8339	77	Phe	GAA
12	8346-8421	76	Glu	TTC
13	8482-8556	75	His	GTG
14	8743-8817	75	Thr	TGT

Supplementary Fig. S1

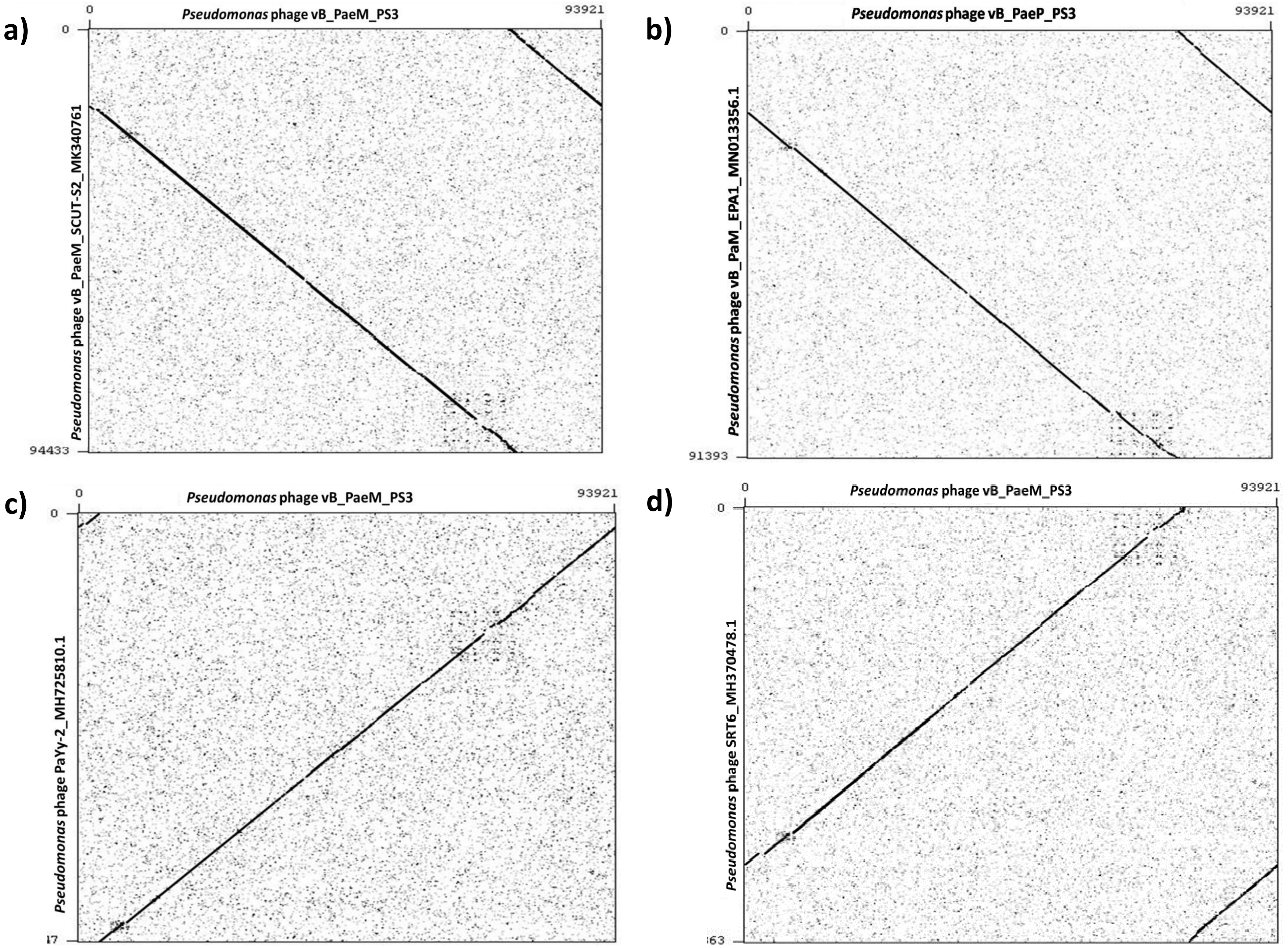

Supplement: Supplementary file 1 — Supplementary file1 (PDF 727 KB) [file 10096_2023_4649_MOESM1_ESM.pdf]
